# The London and Provincial Medical Directory

**Published:** 1847-04

**Authors:** 


### THE LONDON AND PROVINCIAL MEDICAL DIRECTORY. 1847.

In the present edition of this very useful work, we have the Directory for the Country, conjoined with that for London. We think this union highly proper; as the profession is one, wherever the members may be living. This being the first year in which the names of the provincial practitioners are enrolled, the part of the Directory devoted to them is much less perfect than the other. We doubt not, however, from the manifest zeal of the editors, that next year will show a great amendment in this respect. There is an *addition* we would recommend to the notice of the editors, which, we think, would be a very great improvement; it is—to give an alphabetical list of the *provincial towns,* with the surnames of all the medical men resident in each. This would not require much space, as a line or two would suffice for each place; e. g. "Manchester: *Bardsley, Black, Turner, Noble, &c., &c.*" Many must feel that it would often be a great convenience to know the names and qualifications of all the practitioners in any one town.

The plan proposed would effect this readily; as all the names under the head of any town could be successively referred to, in their alphabetical series, in the body of the work.

